# Supplementary material for: Commensal acidification of specific gut regions produces a protective priority effect against enteropathogenic bacterial infection
Source: Appl Environ Microbiol. 2025 Aug 13;91(9):e00707-25. doi: 10.1128/aem.00707-25 (PMC12442404; doi:10.1128/aem.00707-25)
Supplement: Supplemental material — Table S1 and Figure S1. [file aem.00707-25-s0001.pdf]

Supplementary Table S1. Spatial localization of *L. plantarum* and *S. marcescens* in the fly digestive tract. Colonization scoring: 0 = less than 10 cells; 1 = 10 to 100 cells; 2 = 100 to 1000 cells; 3 = more than 1000 cells.

Panel in Figure S1	Crop	Crop duct	Proven-triculus	Posterior A2 & A3 of Midgut	Posterior Midgut	Hindgut	Rectum
B (in Fig 5)	LP: 2<br>SM: 0	LP: 2<br>SM: 1	LP: 2<br>SM: 0	LP: 1<br>SM: 2	LP: 3<br>SM: 0	LP: 2<br>SM: 0	LP: 3<br>SM: 0
C	LP: 1<br>SM: 3	LP: 1<br>SM: 2	LP: 1<br>SM: 0	LP: 0<br>SM: 2	LP: 3<br>SM: 0	LP: 2<br>SM: 0	LP: 3<br>SM: 0
D	LP: 2<br>SM: 1	LP: 2<br>SM: 0	LP: 1<br>SM: 0	LP: 1<br>SM: 2	LP: 3<br>SM: 0	LP: 3<br>SM: 0	No data
E	LP: 0<br>SM: 3	LP: 0<br>SM: 2	LP: 0<br>SM: 0	LP: 0<br>SM: 2	LP: 3<br>SM: 0	LP: 2<br>SM: 0	LP: 2<br>SM: 0
F	LP: 2<br>SM: 0	LP: 2<br>SM: 0	LP: 1<br>SM: 0	LP: 2<br>SM: 0	LP: 3<br>SM: 0	LP: 2<br>SM: 0	LP: 3<br>SM: 0
G	LP: 2<br>SM: 0	LP: 2<br>SM: 0	LP: 1<br>SM: 0	LP: 1<br>SM: 2	LP: 3<br>SM: 0	LP: 1<br>SM: 0	LP: 3<br>SM: 0

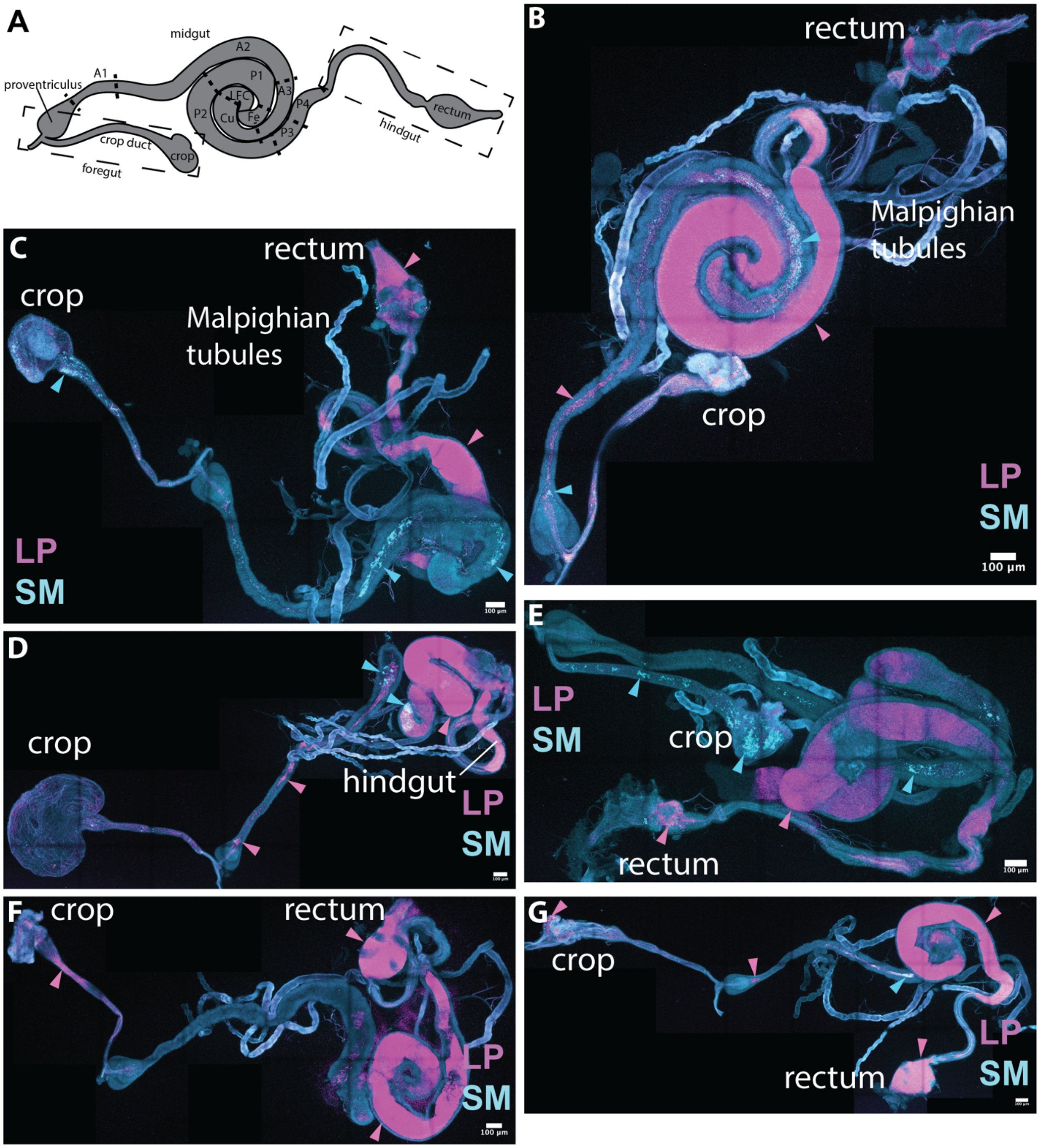

Supplementary Figure S1. Spatial localization of *L. plantarum* and *S. marcescens* in the fly digestive tract. (A) Diagram of the *Drosophila* digestive system with region codes from Marianes & Spradling 2013. (B) Depicted in Figure 5. Maximum intensity projection of a fluorescence micrograph of *Drosophila* gut co-colonized by *L. plantarum* (LP; mCherry-labeled, pink) and *S. marcescens* (SM; mGFP-labeled, blue). (C-G) biological replicate guts prepared as in Figure 5. Labels for crop, hindgut and rectum are to orient readers unfamiliar with fly gut anatomy. Malpighian tubules are part of the renal system and do not hold bacteria but are autofluorescent. The crop is also often autofluorescent when contracted, due to its chitin.

Arrowheads indicate cells of *L. plantarum* (pink) or *S. marcescens* (blue). Scale bars are 100 μm.
